# Supplementary figures and images for: The polyadenylase PAPI is required for virulence plasmid maintenance in pathogenic bacteria
Source: PLoS Pathog. 2025 May 27;21(5):e1012655. doi: 10.1371/journal.ppat.1012655 (PMC12140428; doi:10.1371/journal.ppat.1012655)

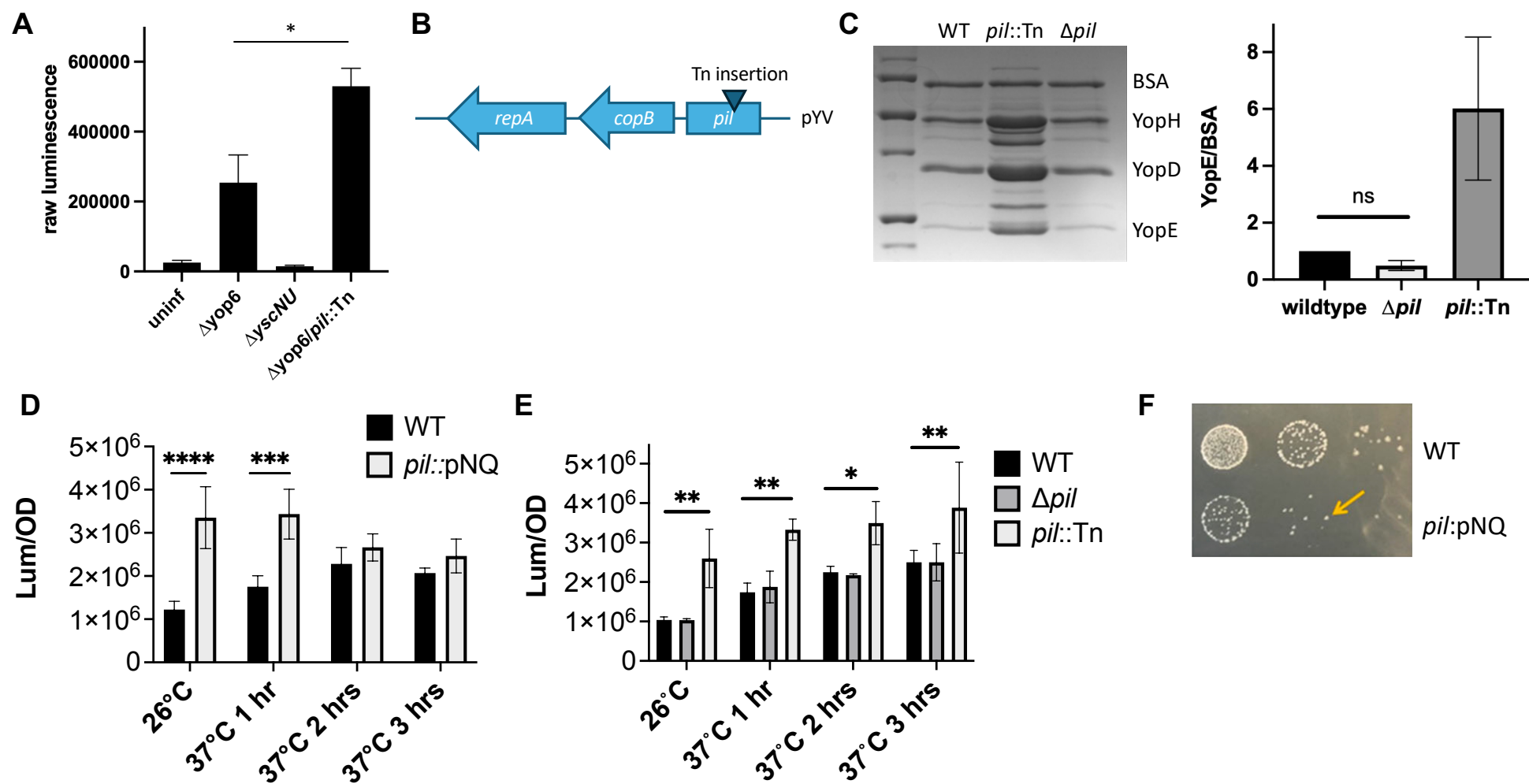

Figure S1

Supplement: S1 Fig — (A) HEK293T cells expressing an NFκB luciferase reporter were left uninfected (uninf) or were infected with an effectorless ∆yop6 strain (∆yopHEMOJ), a ∆yscNU T3SS-deficient strain, or ∆yop6/pil::Tn in the Y. pseudotuberculosis IP2666pIB1 background. Averages of three independent replicates ± standard error the mean are shown (Student t-test, p < 0.04). (B) Schematic representation of the location of pil. The pil locus is located ~123 bp upstream of the copB gene. (C) Wildtype, pil::Tn, and ∆pil IP2666pIB1 were grown at 37°C/low calcium and secreted T3SS cargo proteins (Yops) were precipitated and visualized by Coomassie blue staining. Bovine serum albumin (BSA) serves as a protein precipitation control. Secreted YopE protein levels relative to the BSA control were quantified using densitometry. Graph shows averages ± standard error of the mean for three independent experiments. ns, not significant as determined by ANOVA with Tukey’s multiple comparisons test (p = 0.97). (D-E) Relative pYV PCN was estimated for strains in the YPIII/pIBX background of Y. pseudotuberculosis using a luciferase plasmid copy number assay. For each timepoint, luminescence was measured and normalized to cell density (OD600). Averages ± standard error of the mean for three independent experiments are shown. Statistical significance was calculated using a one-way ANOVA with Bonferroni post-test (**** p < 0.0001, *** p < 0.001, **p < 0.01, * p < 0.05). (F) Serial dilutions for wildtype YPIII/pIBX and the pil::pNQ congenic Y. pseudotuberculosis strains were spotted onto low calcium agar plates for ~16 hours at 37°C before imaging. The yellow arrow indicates an example of a large colony in the pil::pNQ mutant background that represents a candidate suppressor mutant. (PDF) [file ppat.1012655.s001.pdf]

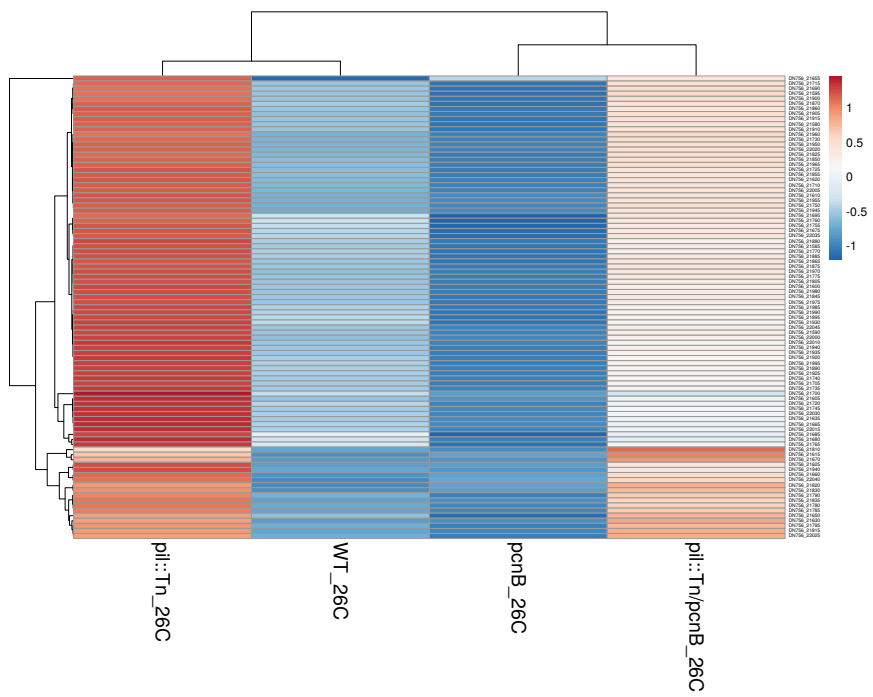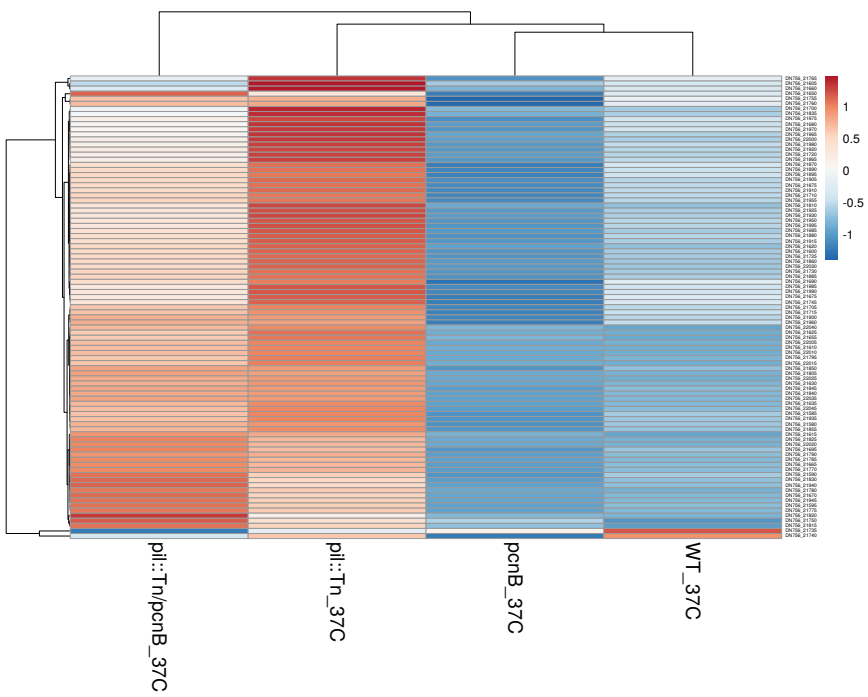

Figure S3

Supplement: S3 Fig — Heat maps displaying relative expression of pYV-encoded genes from RNA-seq analysis of Y. pseudotuberculosis IP2666pIB1 strains grown at 26°C or 37°C/low calcium. (PDF) [file ppat.1012655.s003.pdf]

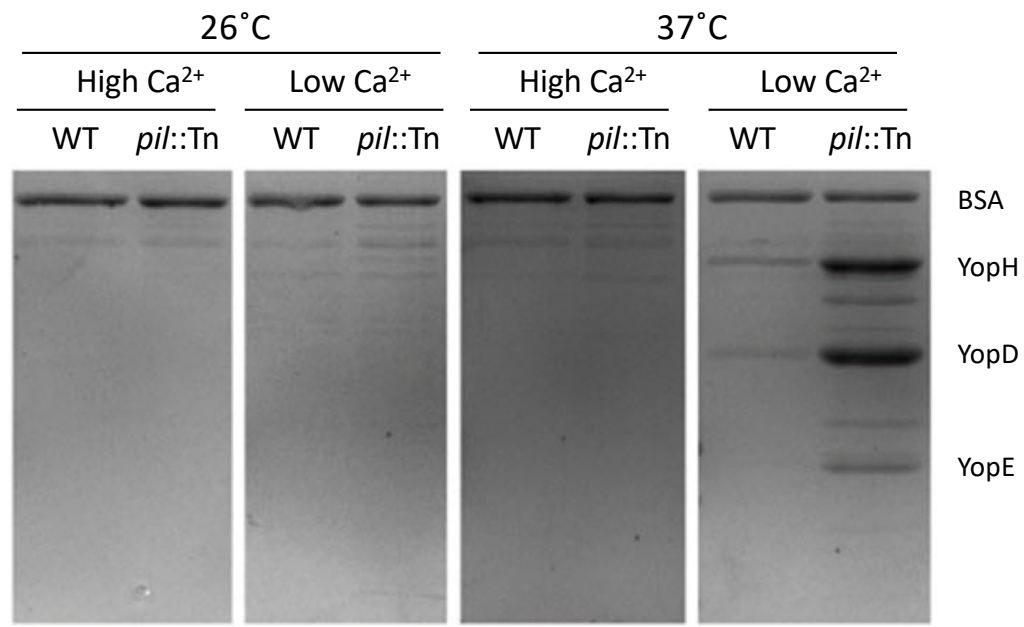

Figure S4

Supplement: S4 Fig — Strains were grown in regular LB or low calcium LB media, at either 26°C or at 37°C. Secreted proteins in the supernatant were precipitated and visualized by SDS-PAGE and Coomassie blue staining. Bovine serum albumin (BSA) was added as a protein precipitation control. Data shown is representative data of three biological replicates. (PDF) [file ppat.1012655.s004.pdf]

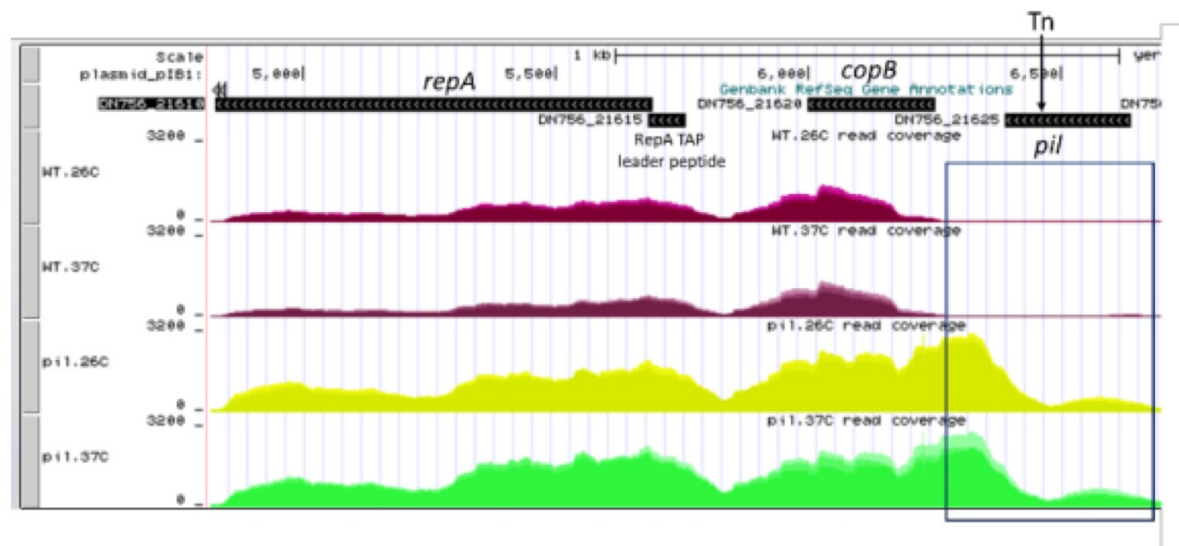

Figure S5

Supplement: S5 Fig — Reads from RNA-seq analysis mapped to the cop-rep locus and adjacent pil locus from wildtype and pil::Tn (pil) Y. pseudotuberculosis IP2666pIB1 grown at 26°C or 37°C/low calcium. The putative pil locus is indicated by a black box and the approximate location of the Tn insertion is indicated with an arrow. (PDF) [file ppat.1012655.s005.pdf]

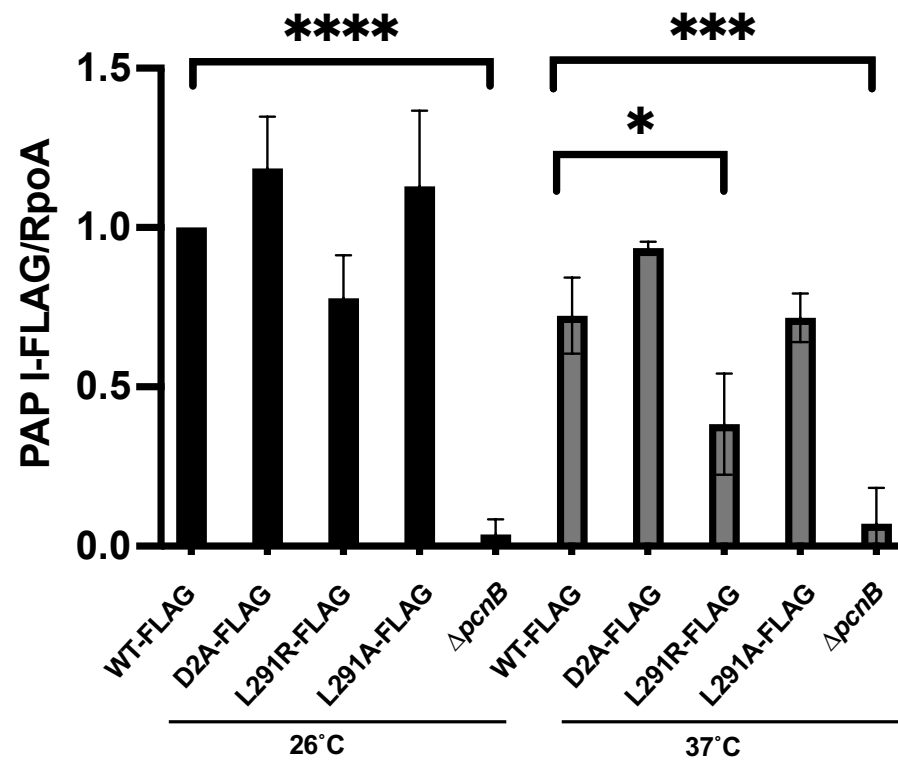

Figure S7

Supplement: S7 Fig — Averages ± standard error of the mean are shown from two independent replicates for the D2A mutant and three independent replicates for the remaining strains. Fig 4A shows one of the replicates. Statistical significance was calculated using a one-way ANOVA with Dunnett’s multiple comparisons test (**** p < 0.0001, *** p = 0.0002, * p < 0.02). (PDF) [file ppat.1012655.s007.pdf]

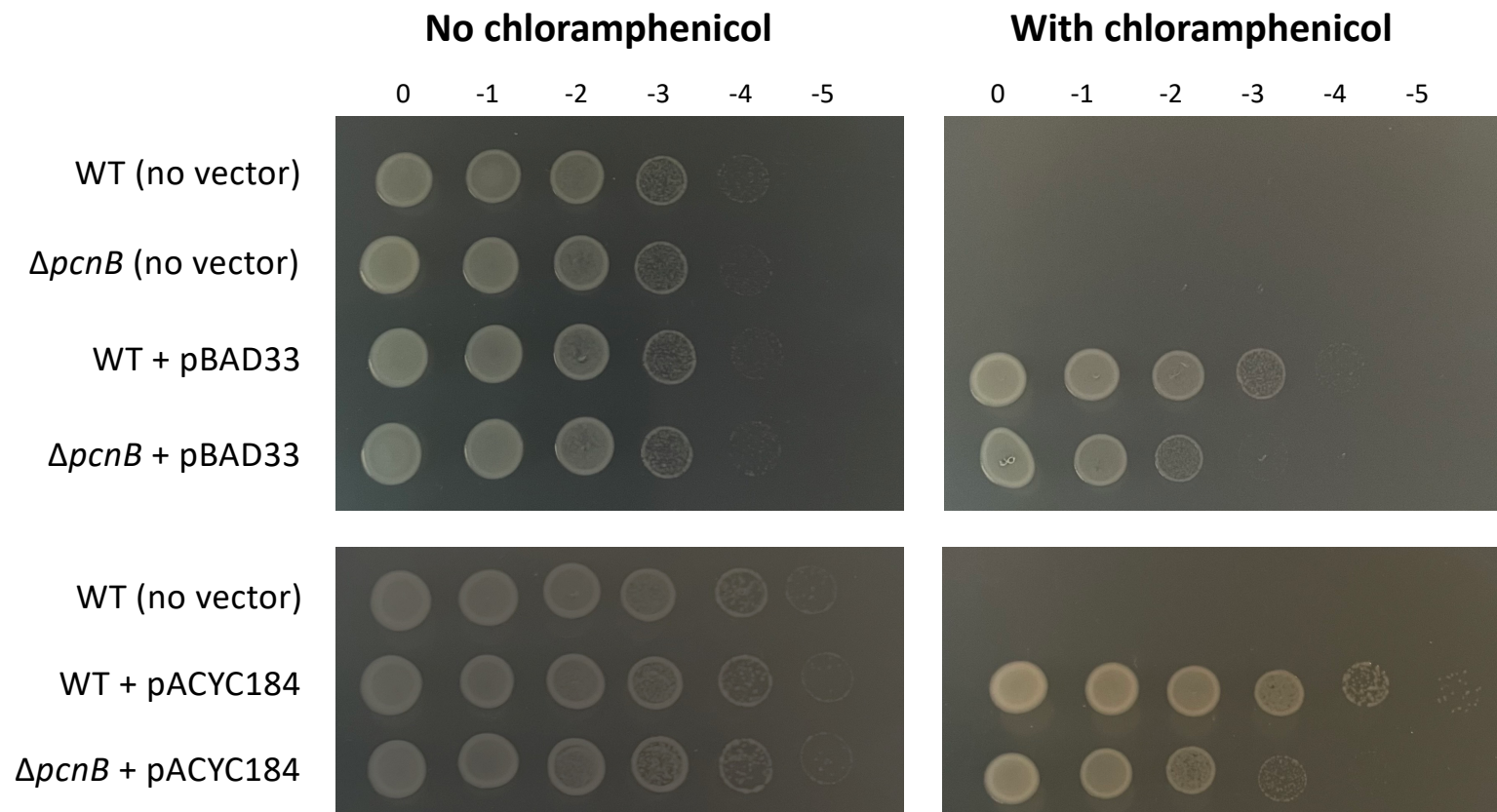

Figure S8

Supplement: S8 Fig — Wildtype and ∆pcnB Y. pseudotuberculosis IP2666pIB1 harboring either pBAD33 or pACYC184 were grown overnight in LB supplemented with 20 µg/mL chloramphenicol, and the overnight cultures spotted onto LB agar lacking or containing chloramphenicol to assess plasmid retention. Plates were incubated at 26°C for ~16 hours before imaging. Each experiment was carried out for a total of two independent experiments. (PDF) [file ppat.1012655.s008.pdf]

**A**

|                     | 26°C | 37°C |
|---------------------|------|------|
| WT                  | 100% | 94%  |
| $\Delta pcnB$       | 89%  | 39%  |
| WT-3                | 100% | 94%  |
| WT-5                | 100% | 67%  |
| $\Delta pcnB$ -2-9  | 85%  | 16%  |
| $\Delta pcnB$ -2-10 | 92%  | 25%  |
| $\Delta pcnB$ -3-1  | 83%  | 8%   |
| $\Delta pcnB$ -3-2  | 91%  | 23%  |

**B**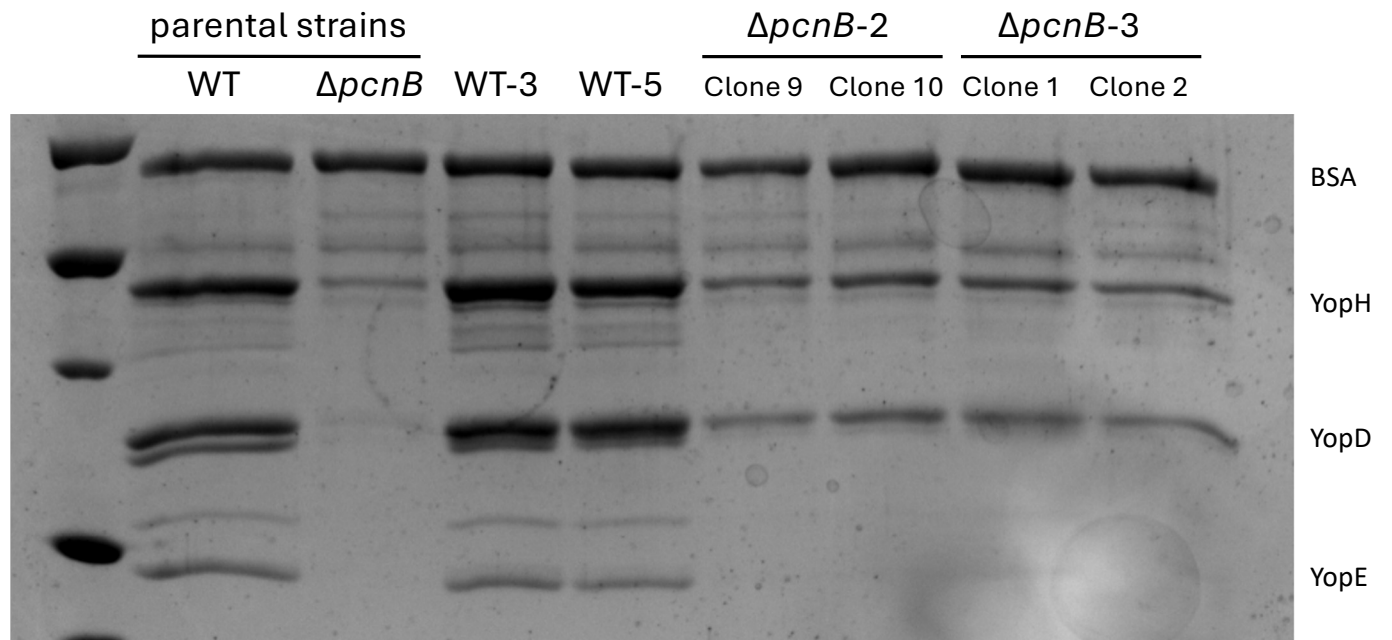

Figure S9

Supplement: S9 Fig — Independent colony forming units from infected mouse livers were isolated and assessed for their ability to retain pYV and carry out T3SS activity. WT-3 and WT-5 are wildtype IP2666pIB1-derived isolates from two different mice. ∆pcnB-2–9, ∆pcnB-2–10, ∆pcnB-3–1, and ∆pcnB-3–2 are ∆pcnB-derived isolates from two different mice. (A) Liver isolates and their parental strains were incubated overnight at 26˚C in LB or 37˚C in LB/low calcium media. Colonies were patched onto low calcium media containing Congo red to induce T3SS activity and assess the percentage of colonies that retained pYV (i.e.,-red colonies). (B) Liver isolates and their parental strains were incubated in LB low calcium media at 37˚C, and secreted proteins visualized by Coomassie blue staining. Bovine serum albumin (BSA) was added as a protein precipitation control. Data shown is representative of two independent experiments. (PDF) [file ppat.1012655.s009.pdf]

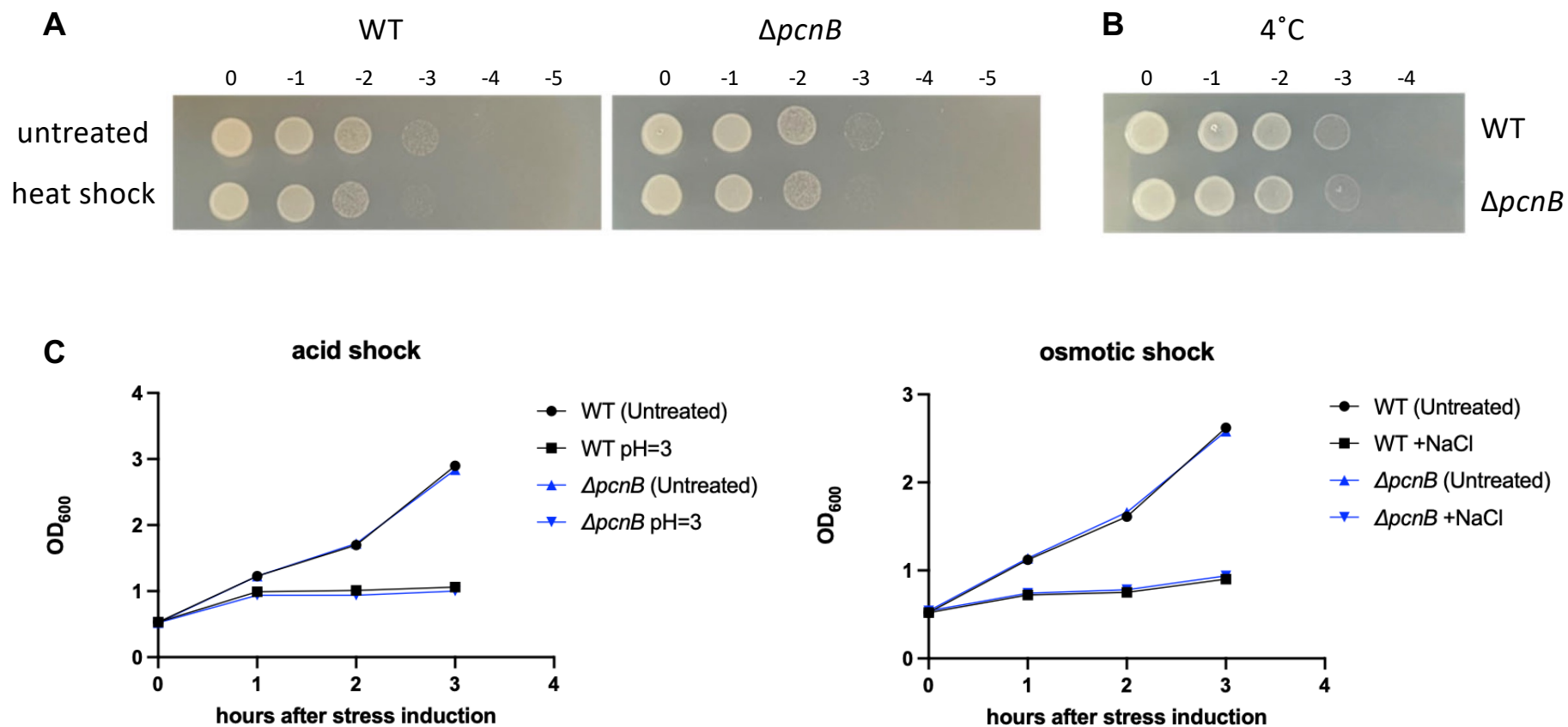

Figure S10

Supplement: S10 Fig — (A) Wildtype and ∆pcnB Y. pseudotuberculosis IP2666pIB1 cultures were grown to mid-log phase at 26°C before shifting to 42°C for 1 hour to induce heat shock. Spot dilutions were plated onto LB plates and incubated at 26°C for ~16 hours before imaging. (B) Strains in the IP2666pIB1 background were grown at 26°C overnight (stationary phase) before diluting and spotting into LB plates. Plates were incubated at 26°C for 4 hours before transferring to 4°C and allowing growth for ~2 days prior to imaging. (C) Wildtype and ∆pcnB Y. pseudotuberculosis IP2666pIB1 were grown at 37°C (low calcium) to mid-log phase before inducing stress. Growth was assayed by plating spot dilutions following stress exposure. Stress was induced by adjusting media to pH = 3.0 to induce acid shock or by addition of 800 mM NaCl2 to induce osmotic shock. (PDF) [file ppat.1012655.s010.pdf]

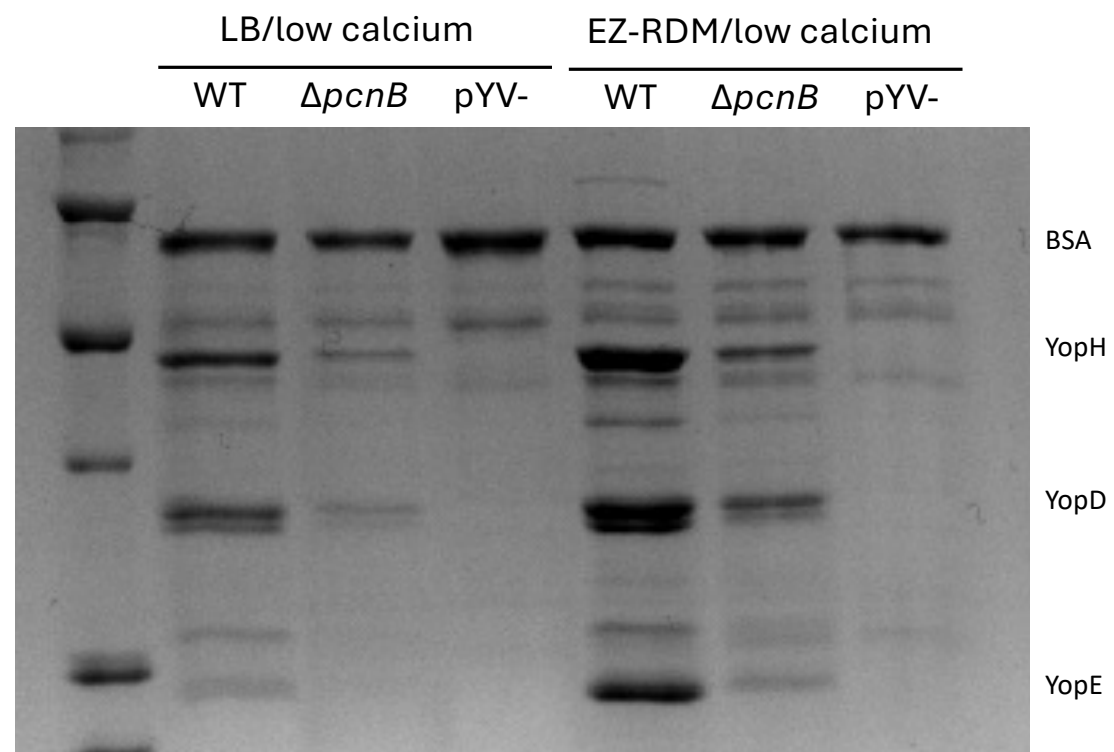

Figure S12

Supplement: S12 Fig — Y. pseudotuberculosis IP2666pIB1 wildtype, ∆pcnB, and pYV- strains were grown in either low calcium LB or low calcium EZ-RDM media. The pYV- strain cured of pYV was used as a negative control [110]. Secreted proteins were visualized by Coomassie blue staining. Bovine serum albumin (BSA) was added as a protein precipitation control. Data shown is representative of two independent experiments. (PDF) [file ppat.1012655.s012.pdf]
