# Supplementary material for: The polyadenylase PAPI is required for virulence plasmid maintenance in pathogenic bacteria
Source: PLoS Pathog. 2025 May 27;21(5):e1012655. doi: 10.1371/journal.ppat.1012655 (PMC12140428; doi:10.1371/journal.ppat.1012655)
Supplement: S6 Fig — (A) Distribution of detected fluorescent ParB-msfGFP spots in the parental or ∆pcnB cells in low-fluorescence state in Fig 3B (26ºC, left). The fraction occupancy of each bin is annotated above each bar. The total number of cells in the low-fluorescence state is noted in each graph. The number of cells in each bin was normalized to the total number of cells for the frequency. (B) Same as (A) but for the cells in low-fluorescence state under 37ºC/low calcium condition. (PDF) [file ppat.1012655.s006.pdf]

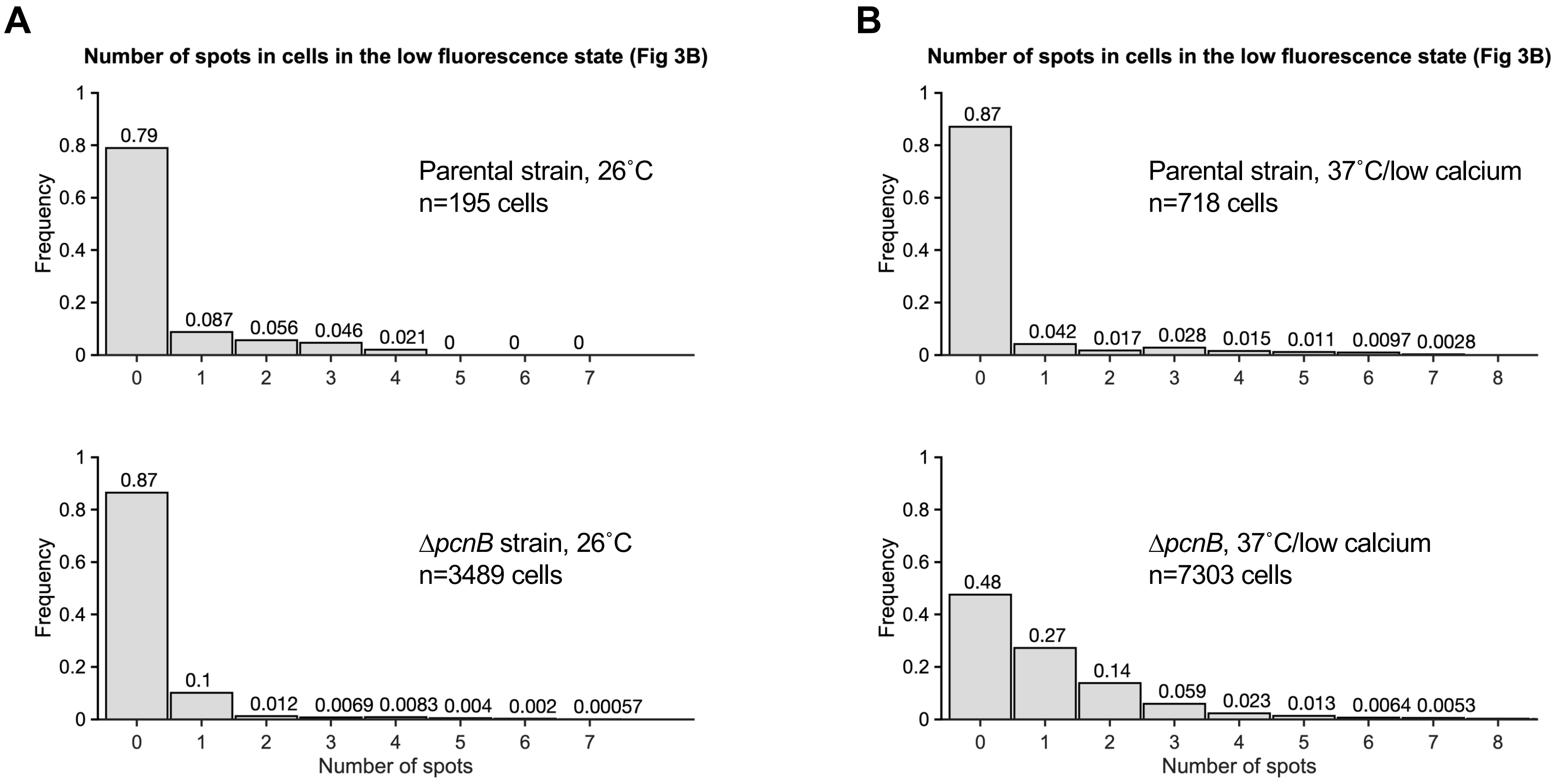
A
Number of spots in cells in the low fluorescence state (Fig 3B)
Parental strain, 26˚C
n=195 cells
0.79
0.087
0.056
0.046
0.021
0
0
0
ΔpcnB strain, 26˚C
n=3489 cells
0.87
0.1
0.012
0.0069
0.0083
0.004
0.002
0.00057
Frequency
Number of spots
B
Number of spots in cells in the low fluorescence state (Fig 3B)
Parental strain, 37˚C/low calcium
n=718 cells
0.87
0.042
0.017
0.028
0.015
0.011
0.0097
0.0028
ΔpcnB, 37˚C/low calcium
n=7303 cells
0.48
0.27
0.14
0.059
0.023
0.013
0.0064
0.0053
Frequency
Number of spots

Figure S6
