# Supplementary material for: The polyadenylase PAPI is required for virulence plasmid maintenance in pathogenic bacteria
Source: PLoS Pathog. 2025 May 27;21(5):e1012655. doi: 10.1371/journal.ppat.1012655 (PMC12140428; doi:10.1371/journal.ppat.1012655)
Supplement: S11 Fig — PAP I sequences from Y. pseudotuberculosis IP2666pIB1 and Y. pestis KIM5. (PDF) [file ppat.1012655.s011.pdf]

```
Y.pseudotuberculosis(IP2666)      MFTRVANFCRKVLIREDKTVRDDKARKDKVPGEDNVARKERRPARAHTGRKGHAVSSSEQ  60
Y.pestis(Kim5)                    MFTRVANFCRKVLIREDKTVRDDKARKDKVPGEDNVARKERRPARAHTGRKGHAVSSSEQ  60
                                  ************************************************************

Y.pseudotuberculosis(IP2666)      RQMAIIPRDQHNISRRDISDNALKVLYRLNKSGYEAYLVGGGVRDLLLGRKPKDFDITTS  120
Y.pestis(Kim5)                    RQMAIIPRDQHNISRRDISDNALKVLYRLNKSGYEAYLVGGGVRDLLLGRKPKDFDITTS  120
                                  ************************************************************

Y.pseudotuberculosis(IP2666)      ATPEQVRKLFRNCRLVGRRFRLAHVMFGPEIIEVATFRGHHEQQQAEDSDKNSSQQAQNG  180
Y.pestis(Kim5)                    ATPEQVRKLFRNCRLVGRRFRLAHVMFGPEIIEVATFRGHHEQQQAEDSDKNSSQQAQNG  180
                                  ************************************************************

Y.pseudotuberculosis(IP2666)      MLLRDNIFGSIEDDAQRRDFTINSLYYGISDFALRDYTGGLRDLKEGIIRLIGDPETRYR  240
Y.pestis(Kim5)                    MLLRDNIFGSIEDDAQRRDFTINSLYYGISDFALRDYTGGLRDLKEGIIRLIGDPETRYR  240
                                  ************************************************************

Y.pseudotuberculosis(IP2666)      EDPVRMLRAVRFAAKLDMSISPETAEPIPRLASLLREIPPARLFEESLKLLQSGYGYKTY  300
Y.pestis(Kim5)                    EDPVRMLRAVRFAAKLDMSISPETAEPIPRLASLLREIPPARLFEESLKLLQSGYGYKTY  300
                                  ************************************************************

Y.pseudotuberculosis(IP2666)      LKLCEYQLFQPLFPLIARNFTEQHDSPMERILVQVLKNTDHRLHNDQRVNPAFLFAAMLW  360
Y.pestis(Kim5)                    LKLCEYQLFQPLFPLIARNFTEQHDSPMERILVQVLKNTDHRLHNDQRVNPAFLFAAMLW  360
                                  ************************************************************

Y.pseudotuberculosis(IP2666)      YPLIEHAQKLTQESGLAYYDAFALAMNDVLDEECRSLAIPKRITSLVRDIWLLQLRLSRR  420
Y.pestis(Kim5)                    YPLIEHAQKLTQESGLAYYDAFALAMNDVLDEECRSLAIPKRITSLVRDIWLLQLRLSRR  420
                                  ************************************************************

Y.pseudotuberculosis(IP2666)      QGKRAHKLMEHPKFRAAYDLLLLRAEVEKNHELQRLAQWWGEFQEATPTQQKSMLNTLGA  480
Y.pestis(Kim5)                    QGKRAHKLMEHPKFRAAYDLLLLRAEVEKNHELQRLAQWWGEFQEATPTQQKSMLNTLGA  480
                                  ************************************************************

Y.pseudotuberculosis(IP2666)      DPAPRRSRPRRPRKPVPRKEGV        502
Y.pestis(Kim5)                    DPAPRRSRPRRPRKPVPRKEGV        502
                                  **********************
```

Figure S11
