# Supplementary material for: The polyadenylase PAPI is required for virulence plasmid maintenance in pathogenic bacteria
Source: PLoS Pathog. 2025 May 27;21(5):e1012655. doi: 10.1371/journal.ppat.1012655 (PMC12140428; doi:10.1371/journal.ppat.1012655)
Supplement: S1 Table — (DOCX) [file ppat.1012655.s013.docx]

**Table S1. Phenotypic categories and mutations identified in suppressor screen**

| Strain: | PCN 26°C^1^ | PCN 37°C^1^ | T3SS activity^3^ | Gene name | Identified Mutation: | Phenotypes: |
| --- | --- | --- | --- | --- | --- | --- |
| pil::pNQ  (parental) | High^2^ | High ^2^ | High ^2^ |  | N/A |  |
| IS0002 | No Change | No change | No Activity | *yscI* | TGA 🡪 GGA mutation abolishing YscI stop codon | Predicted to decrease or abolish translation of YscI |
| IS0004 | No Change | Lower | Lower | *repA* | G 🡪 A mutation in *prepA* -35 region | Predicted to decrease  p*repA* promoter firing |
| IS0006 | Lower | Lower | Lower | *pcnB* | L291R mutation in PAP I | Mutation lowers PAP I protein levels at 37°C |
| IS0007 | No Change | No Change | No Activity | *yscN* | K175E mutation abolishing the essential lysine in walker box A of YscN | Mutation predicted to impair YscN ATP binding and hydrolysis |
| IS0008 | Lower | Lower | Lower | *pcnB* | L291R mutation in PAP I | Mutation lowers PAP I protein levels at 37°C |
| IS0016 | Lower | Lower | Lower | *rpoA* | A272E in RNA Pol alpha factor | Mutation may impact RNA Pol binding to some promoters [1] |

^1^ Relative pYV PCN was determined using a luciferase PCN assay with luminescence
 normalized to OD_600_.

^2^ Changes in pYV PCN and T3SS activity relative to the parental strain. The parental strain
 for *pil::pNQ* is wildtype *Y. pseudotuberculosis* YPIII/pIBX and the parental strain for all
 suppressor isolates shown is *pil::pNQ* YPIII/pIBX.

^3^ T3SS activity was determined via a secretion assay of cells grown at 37°C in low calcium
 media.

1. Belin D, Costafrolaz J, Silva F. AraC Functional Suppressors of Mutations in the C-Terminal Domain of the RpoA Subunit of the *Escherichia coli* RNA Polymerase. Microorganisms. 2024;12(9). Epub 20240923. doi: 10.3390/microorganisms12091928. PubMed PMID: 39338602; PubMed Central PMCID: PMCPMC11434276.
