# Supplementary material for: The polyadenylase PAPI is required for virulence plasmid maintenance in pathogenic bacteria
Source: PLoS Pathog. 2025 May 27;21(5):e1012655. doi: 10.1371/journal.ppat.1012655 (PMC12140428; doi:10.1371/journal.ppat.1012655)
Supplement: S2 Table — (DOCX) [file ppat.1012655.s014.docx]

**Table S2. *Y. pseudotuberculosis* strains used in this study**

| Strain | Background | Mutation(s) | Ref |
| --- | --- | --- | --- |
| Wildtype (WT) | IP2666pIB1 | Naturally lacks full-length YopT | [1] |
| *pil::Tn* | IP2666pIB1 | Tn5 insertion in *pil,* wildtype background | This work |
| *∆pcnB* | IP2666pIB1 | *∆pcnB* | This work |
| *pil*::Tn*/*∆*pcnB* | IP2666pIB1 | *∆pcnB, pil::Tn* background | This work |
| PAP I^His^ | IP2666pIB1 | PAP I with C-6xHis tag | This work |
| PAP I^D2A^ | IP2666pIB1 | D114A+D116A mutations in PAP I (C-6xHis) | This work |
| PAP I^L291R^ | IP2666pIB1 | L291R mutation in PAP I (C-6xHis) | This work |
| PAP I^L291A^ | IP2666pIB1 | L291A mutation in PAP I (C-6xHis) | This work |
| PAP I^FLAG^ | IP2666pIB1 | PAP I with C-3xFLAG tag | This work |
| PAP I^D2A-FLAG^ | IP2666pIB1 | D114A+D116A mutations in PAPI(C-3xFLAG) | This work |
| PAP I^L291R-FLAG^ | IP2666pIB1 | L291R mutation in PAP I (C-3xFLAG) | This work |
| PAP I^L291A-FLAG^ | IP2666pIB1 | L291A mutation in PAP I (C-3xFLAG) | This work |
| *ΔyopHEMOJ* | IP2666pIB1 | *ΔyopHEMOJ* | [2] |
| *ΔyscNU* | IP2666pIB1 | *ΔyscNU* | [3] |
| pYV- | IP2666 | Cured of pYV | [3] |
| *pil*::Tn | IP2666pIB1 | Tn5 insertion in *pil, ∆yopHEMOJ* background | This work |
| Wildtype (WT) | YPIII pIBX | Naturally lacks full-length YopT; pIBX is pYV encoding two copies of the *luxCDABE* operon and a kanamycin resistance gene | [4] |
| *pil*::pNQ | YPIII pIBX | pNQ insertion in *pil* | This work |
| *∆pcnB* | YPIII pIBX | *∆pcnB* | This work |
| ParB-msfGFP | YPIII pIBX | ParB-C-msfGFP | This work |
| ParB-msfGFP/ ∆*pcnB* | YPIII pIBX | *∆pcnB* ParB-C-msfGFP | This work |
| PAP I^His^ | YPIII pIBX | PAP I with C-6xHis tag | This work |
| PAP I^D2A^ | YPIII pIBX | D114A+D116A mutations in PAP I (C-6xHis) | This work |
| PAP I^L291R^ | YPIII pIBX | L291R mutation in PAP I (C-6xHis) | This work |
| PAP I^L291A^ | YPIII pIBX | L291A mutation in PAP I (C-6xHis) | This work |
| ∆*ipaH2.5::tet^R^* | M90T | ∆*ipaH2.5* | [5] |
| ∆*ipaH2.5/* ∆*pcnB* | M90T | ∆*ipaH2.5*∆*pcnB* | This work |
| VP- | M90T | BS176 | [6] |

1. Bliska JB, Guan KL, Dixon JE, Falkow S. Tyrosine phosphate hydrolysis of host proteins by an essential *Yersinia* virulence determinant. Proc Natl Acad Sci U S A. 1991;88(4):1187-91. doi: 10.1073/pnas.88.4.1187. PubMed PMID: 1705028; PubMed Central PMCID: PMCPMC50982.

2. Auerbuch V, Golenbock DT, Isberg RR. Innate immune recognition of *Yersinia pseudotuberculosis* type III secretion. PLoS Pathog. 2009;5(12):e1000686. Epub 20091204. doi: 10.1371/journal.ppat.1000686. PubMed PMID: 19997504; PubMed Central PMCID: PMCPMC2779593.

3. Balada-Llasat JM, Mecsas J. *Yersinia* has a tropism for B and T cell zones of lymph nodes that is independent of the type III secretion system. PLoS Pathog. 2006;2(9):e86. doi: 10.1371/journal.ppat.0020086. PubMed PMID: 16948531; PubMed Central PMCID: PMCPMC1557584.

4. Fahlgren A, Avican K, Westermark L, Nordfelth R, Fallman M. Colonization of cecum is important for development of persistent infection by *Yersinia pseudotuberculosis*. Infect Immun. 2014;82(8):3471-82. Epub 20140602. doi: 10.1128/IAI.01793-14. PubMed PMID: 24891107; PubMed Central PMCID: PMCPMC4136198.

5. Sidik S, Kottwitz H, Benjamin J, Ryu J, Jarrar A, Garduno R, et al. A *Shigella flexneri* virulence plasmid encoded factor controls production of outer membrane vesicles. G3 (Bethesda). 2014;4(12):2493-503. Epub 20141105. doi: 10.1534/g3.114.014381. PubMed PMID: 25378474; PubMed Central PMCID: PMCPMC4267944.

6. Sansonetti PJ, Mounier J. Metabolic events mediating early killing of host cells infected by *Shigella flexneri*. Microb Pathog. 1987;3(1):53-61. doi: 10.1016/0882-4010(87)90037-4. PubMed PMID: 2848171.
