# Supplementary material for: The polyadenylase PAPI is required for virulence plasmid maintenance in pathogenic bacteria
Source: PLoS Pathog. 2025 May 27;21(5):e1012655. doi: 10.1371/journal.ppat.1012655 (PMC12140428; doi:10.1371/journal.ppat.1012655)
Supplement: S3 Table — (DOCX) [file ppat.1012655.s015.docx]

**Table S3. Primers used in this study**

| **Name** | **Primer Sequence** | **Ref** |
| --- | --- | --- |
| **Primers used to make pCVD442::*∆pcnB*** | |  |
| FpcnB_500up_pCVD | caacataaaggtgaatcccatatgAGAAGATTTTATTATCCGTCG | This work |
| RpcnB_500up_pCVD | ggtaaaattaAATGGTACACCTCGATAG | This work |
| FpcnB_500d_pCVD | gtgtaccattTAATTTTACCATGATCCGGGTC | This work |
| RpcnB_500d_pCVD | acctggcacggctgggacggaagtcTGAGCCGGCGATATTACC | This work |
| **Primers used to make pCVD442::PAPI^His^** | |  |
| FpcnB_pET28 | agtggtggtggtggtggtgctcgagTACCCCTTCTTTACGGGG | This work |
| RpcnB_pET28 | actttaagaaggagatataccatggATTTTTACCCGAGTAGCC | This work |
| FpcnB_500up_His | gaggtgtaccATTTTTACCCGAGTAGCCAATTTC | This work |
| RpcnB_500up_His | catggtaaaaTCAGTGGTGGTGGTGGTG | This work |
| FpET_pB_His_300d | caacataaaggtgaatcccatatgAGGGGAATAAGCTCTCCAAG | This work |
| RpET_pB_His_300d | gggtaaaaatGGTACACCTCGATAGTGG | This work |
| FpcnB_300d_pCVD | ccaccactgaTTTTACCATGATCCGGGTC | This work |
| RpcnB_300d_pCVD | tgacagtctccggaagacggTGAGCCGGCGATATTACC | This work |
| **Primers used to make pCVD442::PAPI^FLAG^** | |  |
| FpcnB_FLAG | caacataaaggtgaatcccaTGAAAAATACCGACCACC | This work |
| RpcnB_FLAG | ctttgtagtcTACCCCTTCTTTACGGGG | This work |
| FpET_FLAG_pcnB | agaaggggtaGACTACAAAGACCATGACG | This work |
| RpET_FLAG_pcnB | ggtaaaattaCATATGGTACCAGCTGCAG | This work |
| FpcnB_500d_pCVD | gtaccatatgTAATTTTACCATGATCCGGGTC | This work |
| RpcnB_500d_pCVD | tgacagtctccggaagacggTGAGCCGGCGATATTACC | This work |
| **Primers used to make pCVD442::ParB-msfGFP** | |  |
| FparB_GFP | caacataaaggtgaatcccatatgTTGCTAATGAGTATCGTC | This work |
| RparB_GFP | cgccttttgaCAAAGAATGTTCCTTTGC | This work |
| FpKD_GFP_parB | acattctttgTCAAAAGGCGAAGAACTTTTTAC | This work |
| RpKD_GFP_parB | tattcaggcaTTATTTATACAATTCATCCATTCCATGAGTGAT TCCTGCCGCAGTGACAAATTC | This work |
| FparB_500d_pCVD | gtataaataaTGCCTGAATAAGATCAGAAC | This work |
| RparB_500d_pCVD | tgacagtctccggaagacggAATCTCCCTAAAGCTATCAC | This work |
| **Q5 mutagenesis primers used to introduce L291R mutation** | |  |
| FpB_L291R_pCVD | ACTCAAGCTGaggCAATCCGGCTAC | This work |
| RpB_L291R_pCVD | GACTCCTCAAACAGGCGG | This work |
| **Q5 mutagenesis primers used to introduce L291A mutation** | |  |
| FpB_L291A_pCVD | ACTCAAGCTGgcgCAATCCGGCTAC | This work |
| RpB_L291A_pCVD | GACTCCTCAAACAGGCGG | This work |
| **Q5 mutagenesis primers used to introduce D114A + D116A (D2A) mutations** | | |
| FpB_D2A_pCVD | tcgctATCACCACCAGCGC | This work |
| RpB_D2A_pCVD | aagcTTTGGGTTTTCTGCCC | This work |
| **Primers used to make pTrc99::PAP I** | | |
| FpcnB_pTrc | atttcacacaggaaacagaccatggATTTTTACCCGAGTAGCCAATTTC | This work |
| RpcnB_pTrc | tgcatgcctgcaggtcgactctagaCAGCGCGATATAGACCCG | This work |
| **Primers used for ddPCR** | | |
| F_Chrom_ddPCR | CCTCACCGATACCGAACGAG | [1] |
| R_Chrom_ddPCR | GTCAGCAGGATAGGGCTACC | [1] |
| F_pYV_ddPCR | CTCTTTGACCTCGGCTTGAG | [1] |
| R_pYV_ddPCR | CGCAGCCGTTAGGACAAATG | [1] |
| **Primers used to make ∆*ipaH2.5*∆*pcnB Shigella*** | | |
| pcnb_F | ggcagaagcacactggcagg | This work |
| pcnb_R | gtggtccccagcgttcagc | This work |
| pcnB_R_op | gacttcacgcaacgtctcccc | This work |
| K2_wanner | cggtgccctgaatgaactgc | [2] |

1. Schneiders S, Hechard T, Edgren T, Avican K, Fallman M, Fahlgren A, et al. Spatiotemporal Variations in Growth Rate and Virulence Plasmid Copy Number during *Yersinia pseudotuberculosis* Infection. Infect Immun. 2021;89(4). Epub 20210317. doi: 10.1128/IAI.00710-20. PubMed PMID: 33495272; PubMed Central PMCID: PMCPMC8090943.

2. Datsenko KA, Wanner BL. One-step inactivation of chromosomal genes in *Escherichia coli* K-12 using PCR products. Proc Natl Acad Sci U S A. 2000;97(12):6640-5. doi: 10.1073/pnas.120163297. PubMed PMID: 10829079; PubMed Central PMCID: PMCPMC18686.
