# Supplementary material for: The polyadenylase PAPI is required for virulence plasmid maintenance in pathogenic bacteria
Source: PLoS Pathog. 2025 May 27;21(5):e1012655. doi: 10.1371/journal.ppat.1012655 (PMC12140428; doi:10.1371/journal.ppat.1012655)
Supplement: S4 Table — (DOCX) [file ppat.1012655.s016.docx]

**Table S4. Plasmids used in this study**

| **Vector/Plasmid** | **Insert** | **Ref** |
| --- | --- | --- |
| pCVD442::empty | None [SacB^+^] | [1] |
| pET28::empty | -C-3xFLAG and -C-6xHis | [2] |
| pKD13-msfGFP | -C-msfGFP | [3] |
| pCVD442:: *∆pcnB* | *∆pcnB* | This work |
| pCVD442::PAP I^His^ | PAP I-C-6xHis | This work |
| pCVD442::PAP I^L291R^ | PAP I^L291R-C-6xHis^ | This work |
| pCVD442::PAP I^L291A^ | PAP I^L291A-C-6xHis^ | This work |
| pCVD442::PAP I^D2A^ | PAP I^D2A-C-6xHis^ | This work |
| pCVD442::PAP I^FLAG^ | PAP I-C-3xFLAG | This work |
| pCVD442::PAP I^L291R-FLAG^ | PAP I^L291R-C-3xFLAG^ | This work |
| pCVD442::PAP I^L291A-FLAG^ | PAP I^L291A-C-3xFLAG^ | This work |
| pCVD442::PAP I^D2A-FLAG^ | PAP I^D2A-C-3xFLAG^ | This work |
| pCVD442::ParB-msfGFP | ParB-C-msfGFP | This work |
| pTrc99A::empty | None [IPTG-inducible T7] | [4] |
| pTrc99::PAP I | PAP I | This work |
| pGFP-uv | GFPuv | [5] |
| pNF06::ccdAB | ccdAB | [6] |

1. Donnenberg MS, Kaper JB. Construction of an eae deletion mutant of enteropathogenic *Escherichia coli* by using a positive-selection suicide vector. Infect Immun. 1991;59(12):4310-7. doi: 10.1128/iai.59.12.4310-4317.1991. PubMed PMID: 1937792; PubMed Central PMCID: PMCPMC259042.

2. Shis DL, Bennett MR. Library of synthetic transcriptional AND gates built with split T7 RNA polymerase mutants. Proc Natl Acad Sci U S A. 2013;110(13):5028-33. Epub 20130311. doi: 10.1073/pnas.1220157110. PubMed PMID: 23479654; PubMed Central PMCID: PMCPMC3612686.

3. Gray WT, Govers SK, Xiang Y, Parry BR, Campos M, Kim S, et al. Nucleoid Size Scaling and Intracellular Organization of Translation across Bacteria. Cell. 2019;177(6):1632-48 e20. doi: 10.1016/j.cell.2019.05.017. PubMed PMID: 31150626; PubMed Central PMCID: PMCPMC6629263.

4. Amann E, Ochs B, Abel KJ. Tightly regulated tac promoter vectors useful for the expression of unfused and fused proteins in *Escherichia coli*. Gene. 1988;69(2):301-15. doi: 10.1016/0378-1119(88)90440-4. PubMed PMID: 3069586.

5. Million-Weaver S, Alexander DL, Allen JM, Camps M. Quantifying plasmid copy number to investigate plasmid dosage effects associated with directed protein evolution. Methods Mol Biol. 2012;834:33-48. doi: 10.1007/978-1-61779-483-4_3. PubMed PMID: 22144351; PubMed Central PMCID: PMCPMC3804865.

6. Jurenas D, Fraikin N, Goormaghtigh F, De Bruyn P, Vandervelde A, Zedek S, et al. Bistable Expression of a Toxin-Antitoxin System Located in a Cryptic Prophage of *Escherichia coli* O157:H7. mBio. 2021;12(6):e0294721. Epub 20211130. doi: 10.1128/mBio.02947-21. PubMed PMID: 34844426; PubMed Central PMCID: PMCPMC8630535.
