# Supplementary material for: The polyadenylase PAPI is required for virulence plasmid maintenance in pathogenic bacteria
Source: PLoS Pathog. 2025 May 27;21(5):e1012655. doi: 10.1371/journal.ppat.1012655 (PMC12140428; doi:10.1371/journal.ppat.1012655)
Supplement: S2 Fig — (A) PAP I protein sequences from E. coli K12, Y. pseudotuberculosis IP2666, and S. flexneri M90T strains were aligned using Clustal Omega. The PAP I head domain (blue), neck domain (green), body domain (purple), and leg domains (yellow) are shown [67]. Residues in boxes represent known catalytic residues from EcPAP and the residue indicated with an arrow represents the residue that was mutated in the L291R pcnB suppressor mutants. (B) Predicted structures of EcPAP and Y. pseudotuberculosis PAP I obtained using AlphaFold Protein Structure Database. The homologous domains were color-coded with AlphaFold Structure Tools, and follow the coloring scheme described in panel A. (C) Residues D114, D116, and L291 are highlighted in red and indicated by red arrows. The N-terminus (upstream of the head domain) is indicated in black. The unstructured C-termini are not shown in the structure predictions. (PDF) [file ppat.1012655.s002.pdf]

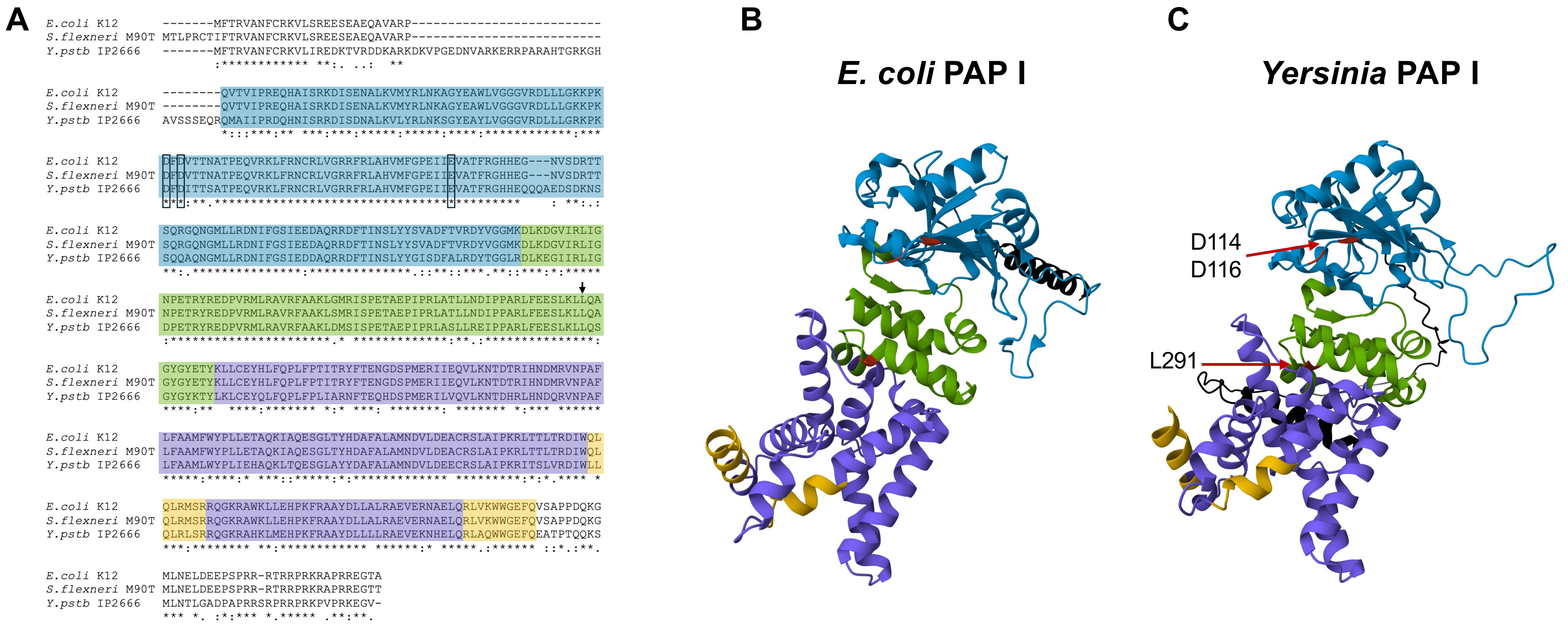
A
E.coli K12 -------MFTRVANFCRKVLSREESEAEQAVARP--------------------------
S.flexneri M90T MTLPRCTIFTRVANFCRKVLSREESEAEQAVARP--------------------------
Y.pstb IP2666 -------MFTRVANFCRKVLIREDKTVRDDKARKDKVPGEDNVARKERRPARAHTGRKGH
:************ **:. ..: **
E.coli K12 --------QVTVIPREQHAISRKDISENALKVMYRLNKAGYEAWLVGGGVRDLLLGKKPK
S.flexneri M90T --------QVTVIPREQHAISRKDISENALKVMYRLNKAGYEAWLVGGGVRDLLLGKKPK
Y.pstb IP2666 AVSSSEQRQMAIIPRDQHNISRRDISDNALKVLYRLNKSGYEAYLVGGGVRDLLLGRKPK
*:::***:** ***:***:*****:*****:****:************:***
E.coli K12 DFDVTTNATPEQVRKLFRNCRLVGRRFRLAHVMFGPEIIEVATFRGHHEG---NVSDRTT
S.flexneri M90T DFDVTTNATPEQVRKLFRNCRLVGRRFRLAHVMFGPEIIEVATFRGHHEG---NVSDRTT
Y.pstb IP2666 DFDITTSATPEQVRKLFRNCRLVGRRFRLAHVMFGPEIIEVATFRGHHEQQQAEDSDKNS
:**.******************************* : **:.:
E.coli K12 SQRGQNGMLLRDNIFGSIEEDAQRRDFTINSLYYSVADFTVRDYVGGMKDLKDGVIRLIG
S.flexneri M90T SQRGQNGMLLRDNIFGSIEEDAQRRDFTINSLYYSVADFTVRDYVGGMKDLKDGVIRLIG
Y.pstb IP2666 SQQAQNGMLLRDNIFGSIEDDAQRRDFTINSLYYGISDFALRDYTGGLRDLKEGIIRLIG
:.*************:***************.::**::***.**::***:*:*****
E.coli K12 NPETRYREDPVRMLRAVRFAAKLGMRISPETAEPIPRLATLLNDIPPARLFEESLKLLQA
S.flexneri M90T NPETRYREDPVRMLRAVRFAAKLSMRISPETAEPIPRLATLLNDIPPARLFEESLKLLQA
Y.pstb IP2666 DPETRYREDPVRMLRAVRFAAKLDMSISPETAEPIPRLASLLREIPPARLFEESLKLLQS
:**********************.* *************:**.:****************:
E.coli K12 GYGYETYKLLCEYHLFQPLFPTITRYFTENGDSPMERIIEQVLKNTDTRIHNDMRVNPAF
S.flexneri M90T GYGYETYKLLCEYHLFQPLFPTITRYFTENGDSPMERIIEQVLKNTDTRIHNDMRVNPAF
Y.pstb IP2666 GYGYKTYLKLCEYQLFQPLFPLIARNFTEQHDSPMERILVQVLKNTDHRLHNDQRVNPAF
****:** ****:******* *:* ***: *******: ******* *:*** ******
E.coli K12 LFAAMFWYPLLETAQKIAQESGLTYHDAFALAMNDVLDEACRSLAIPKRLTTLTRDIWQL
S.flexneri M90T LFAAMFWYPLLETAQKIAQESGLTYHDAFALAMNDVLDEACRSLAIPKRLTTLTRDIWQL
Y.pstb IP2666 LFAAMLWYPLIEHAQKLTQESGLAYYDAFALAMNDVLDEECRSLAIPKRITSLVRDIWLL
*****:****:* ***::*****:*:************* *********:*:*.**** *
E.coli K12 QLRMSRRQGKRAWKLLEHPKFRAAYDLLALRAEVERNAELQRLVKWWGEFQVSAPPDQKG
S.flexneri M90T QLRMSRRQGKRAWKLLEHPKFRAAYDLLALRAEVERNAELQRLVKWWGEFQVSAPPDQKG
Y.pstb IP2666 QLRLSRRQGKRAHKLMEHPKFRAAYDLLLLRAEVEKNHELQRLAQWWGEFQEATPTQQKS
:***** **:************ ******:* *****.:****** ::*.:**.
E.coli K12 MLNELDEEPSPRR-RTRRPRKRAPRREGTA
S.flexneri M90T MLNELDEEPSPRR-RTRRPRKRAPRREGTT
Y.pstb IP2666 MLNTLGADPAPRRSRPRRPRKPVPRKEGV-
*** *. :*:*** *.***** .**:**.
B
E. coli PAP I
C
Yersinia PAP I
D114
D116
L291

Figure S2
